# Bacterial Diversity in Native Heart Valves in Infective Endocarditis

**DOI:** 10.3390/biomedicines13010245

**Published:** 2025-01-20

**Authors:** Anna Sinitskaya, Alexander Kostyunin, Maxim Asanov, Maria Khutornaya, Anastasia Klyueva, Alyona Poddubnyak, Alexey Tupikin, Marsel Kabilov, Maxim Sinitsky

**Affiliations:** 1 Laboratory of Genome Medicine, Research Institute for Complex Issues of Cardiovascular Diseases, 650002 Kemerovo, Russia; 2 Laboratory of Novel Biomaterials, Research Institute for Complex Issues of Cardiovascular Diseases, 650002 Kemerovo, Russia; 3 Institute of Chemical Biology and Fundamental Medicine, Siberian Branch of the Russian Academy of Sciences, 630090 Novosibirsk, Russia

**Keywords:** infective endocarditis, Gram staining, histological analysis, immunohistochemistry, 16S rRNA metabarcoding, taxonomic diversity

## Abstract

**Background:** Infective endocarditis (IE) is an infectious disease caused by the hematogenous dissemination of bacteria into heart valves. Improving the identification of pathogens that cause IE is important to increase the effectiveness of its therapy and reduce the mortality caused by this pathology. **Methods:** Ten native heart valves obtained from IE patients undergoing heart valve replacements were analyzed. Bacterial invasion in the heart valves was studied by Gram staining of histological sections. Histopathological changes accompanied with bacterial invasion were studied by immunohistochemical analysis of pan-leukocyte marker CD45, platelet marker CD41, and neutrophil myeloperoxidase. The taxonomic diversity of the bacteria was analyzed using 16S rRNA metabarcoding. **Results:** Gram staining of the histological sections revealed bacterial cells localized on the atrial surface at the leaflet’s free edge or on the ventricular surface at the leaflet’s base within fibrin deposits in only three of the studied heart valves. Bacterial colonies were co-localized with microthrombi (CD41^+^ cells) containing single leucocytes (CD45^+^ cells), represented by segmented neutrophils. As a result of 16S rRNA metabarcoding, we detected the following bacterial genera: *Pseudomonas* (70% of the studied heart valves), *Roseateles* (60%), *Acinetobacter* (40%), *Sphingomonas* (40%), *Enterococcus* (30%), *Reyranella* (20%), *Sphingobium* (20%), *Streptococcus* (20%), *Agrobacterium* (20%), *Ralstonia* (10%), and *Bacillus* (10%). **Conclusions:** A number of opportunistic microorganisms that could not be detected by routine laboratory tests and were not eliminated during antibiotic therapy were identified in the IE-affected heart valves. The obtained results show the importance of 16S rRNA metabarcoding of heart valves removed due to IE not only as an independent diagnostic method but also as a highly accurate approach that complements routine tests for pathogen identification.

## 1. Introduction

Infective endocarditis (IE) affecting the endocardium is an infectious disease caused by the hematogenous dissemination of bacteria into heart valves, leading to the formation of vegetations [1,2]. IE is characterized by disruption of the heart valve structure, requiring in most cases a radical surgical correction due to the ineffectiveness of antimicrobial therapy [3]. Despite significant advances in early IE diagnosis, the incidence rate of this pathology is currently about 13.8 per 100,000 population [4], with an in-hospital mortality rate ranging from 13% to 25% [5]. Clinical presentation of IE in patients is variable and largely depends on the type of pathogen and affected valve, making the diagnosis of this pathology challenging [6].

It has been shown that IE is mainly caused by streptococci and staphylococci; HACEK organisms (*Haemophilus* spp., *Aggregatibacter actinomycetemcomitans*, *Cardiobacterium hominis*, *Eikenella corrodens*, *Kingella kingae*) and fungi are rarer causes of IE [7,8]. Routine blood culture is the gold standard for pathogen identification in IE patients [9,10], but it can be ineffective in up to 70% of all endocarditis cases due to the sterility of blood cultures [11]. According to the generally accepted clinical recommendations, blood sampling should be performed three times with an interval of at least 6 h to minimize false negative results [12]. To confirm the results of microbiological tests, polymerase chain reaction (PCR) and histopathological analysis of explanted heart valves can be used [11,13,14,15]. Histopathological analysis can also give false negative results when identifying bacteria, but it can be useful to detect some markers of chronic (lymphocytes, neovascularization, etc.) and acute (polymorphonuclear leukocytes) inflammation, as well as fibrin deposits that are co-localized with bacterial agents [13,16]. False negative results of pathogen identification in IE patients by routine tests are attributed to antimicrobial therapy prior to blood sampling, an insufficient number of bacteria to culture, the presence of difficult-cultivated bacteria, etc. [13,17,18], so improving pathogen identification is very important to increase the effectiveness of IE therapy and reduce the mortality caused by this pathology [19].

Nowadays, widely used next-generation sequencing (NGS) allows for the identification of pathogens when routine tests are ineffective [20]. 16S rRNA metabarcoding is an alternative approach that can be used in clinical practice for the identification of bacterial agents at the genus and species levels [21,22]. The 16S rRNA gene can be found in all bacteria and includes highly conserved regions interspersed with variable sequences. The main clinical advantage of this approach is the ability to detect non-viable bacterial DNA even in patients characterized by negative results of routine microbiological tests (e.g., blood culture-negative endocarditis) [23,24].

This study aimed to identify bacterial agents in the native heart valves obtained from IE patients characterized by both negative and positive blood cultures, as well as to study the bacterial invasion-induced histopathological changes in the IE-affected heart valves.

## 2. Materials and Methods

### 2.1. Group Description

A total of 10 native heart valves obtained from IE patients hospitalized at the Research Institute for Complex Issues of Cardiovascular Diseases (Kemerovo, Russia) in 2024 and undergoing elective heart valve replacements were analyzed. IE was confirmed according to the clinical, microbiological (routine blood culture), and echocardiographic (Duke criteria) data [25]. Blood sampling for IE pathogen identification was performed on all patients three times at 6 h intervals one week before surgery. Patients with fever, acute inflammatory diseases, and peripheral arterial embolism were excluded from the study.

The study design was approved by the Institutional Review Board of the Research Institute for Complex Issues of Cardiovascular Diseases (Kemerovo, Russia) (Minutes No. 01, dated 26 January 2024). All patients recruited in the presented study provided written informed consent to participate in the examination. This study complies with the Declaration of Helsinki (ethical principles for medical research involving human participants, amended in 2000) and Good Clinical Practice guidelines.

### 2.2. Histological Analysis

Bacterial invasion in the native heart valves obtained from IE patients was studied by histological analysis. The explanted native heart valves were stored in sterile 0.9% NaCl at 10 °C and immediately transferred to the laboratory. The fragments of damaged heart valve leaflets from their base to their free edge were cut out, placed in Neg-50^TM^ Frozen Section Medium (Epredia, Kalamazoo, MI, USA), and frozen at −25 °C. Next, serial histological sections with a thickness of 6 μm were prepared using a Microm HM 525 Cryostat (Thermo Fisher Scientific, Waltham, MA, USA) and sequentially placed on Polysine^TM^ Adhesion Microscope Slides (Epredia, Kalamazoo, MI, USA). Detection of bacteria was performed using a Gram Stain Kit (Microorganism Stain) (Abcam, Cambridge, UK), according to the manufacture’s recommendations.

The stained sections were analyzed using an AxioImager.A1 microscope (Carl Zeiss MicroImaging GmbH, Jena, Germany) at 400× and 1000× magnification with transmitted light and processed using AxioVision software v.4.8 (Carl Zeiss MicroImaging GmbH, Jena, Germany).

### 2.3. Immunohistochemistry

The histopathological changes accompanied by bacterial invasion in the native heart valves were studied by immunohistochemical analysis. The serial histological sections prepared as described above were fixed in 4% paraformaldehyde at room temperature, followed by washing in phosphate-buffered saline at 50 rpm using a Polymax 1040 shaker (Heidolph Scientific Products GmbH, Schwabach, Germany). Next, the sections were stained using the Novolink^TM^ Polymer Detection System (Leica Biosystems, Nussloch, Germany), according to the manufacture’s protocol, and primary antibodies to the following cellular markers: pan-leukocyte marker CD45 (ab10558, Abcam, Cambridge, UK, 1:2000 dilution), platelet marker CD41 (ab134131, Abcam, Cambridge, UK, 1:2000 dilution), and neutrophil myeloperoxidase (MPO) (ab208670, Abcam, Cambridge, UK, 1:2000 dilution). Briefly, the samples were incubated with the primary antibodies in the dark for 18 h at 4 °C, and the stained sections were placed under a cover glass using Vitrogel mounting medium (BioVitrum, Saint-Petersburg, Russia). The antibodies were diluted in 1% saline solution of bovine serum albumin, and the optimal dilutions were selected by serial staining.

The stained sections were analyzed using an AxioImager.A1 microscope (Carl Zeiss MicroImaging GmbH, Jena, Germany) at 400× magnification with transmitted light and processed using AxioVision software v.4.8 (Carl Zeiss MicroImaging GmbH, Jena, Germany).

### 2.4. 16S rRNA Metabarcoding

Genomic DNA was extracted from the fragments of the IE-affected native heart valve leaflets from their base to their free edge (approximately 50 mg of tissue) stored in sterile 0.9% NaCl using the HostZERO Microbial DNA Kit (Zymo Research, Irvine, CA, USA). The V3–V4 region of the 16S rRNA genes was amplified with the primer pair 343F (5′-CTCCTACGGRRSGCAGCAG-3′) and 806R (5′-GGACTACNVGGGTWTCTAAT-3′) combined with Illumina adapter sequences [26]. PCR amplification was performed in 50 μL reactions containing 0.02 U HS-Taq, 1× PCR buffer, 3.8 mM MgCl_2_ (Biolabmix, Novosibirsk, Russia), 0.2 μM of each forward and reverse primer, 4 ng of template DNA, and 0.2 mM of each dNTP (Life Technologies, Waltham, MA, USA). The thermal cycling conditions were as follows: initial denaturation at 95 °C for 5 min, followed by 30 cycles of 95 °C for 15 s, 62 °C for 15 s, and 72 °C for 30 s, with a final extension at 72 °C for 5 min. A total of 200 ng PCR product from each sample (mix of three technical replicates) was pooled together and purified using the MinElute Gel Extraction Kit (Qiagen, Hilden, Germany). The 16S libraries were sequenced with Reagent Kit v3 (2 × 300 bp) on MiSeq (Illumina, San Diego, CA, USA) at the SB RAS Genomics Core Facility (Institute of Chemical Biology and Fundamental Medicine, Novosibirsk, Russia).

Raw sequences were analyzed with UPARSE pipeline [27] using Usearch v11.0.667. The UPARSE pipeline included merging of paired reads, read quality filtering, length trimming, merging of identical reads (dereplication), discarding singleton reads, removing chimeras, and OTU (operational taxonomic unit) clustering using the UPARSE-OTU algorithm [28]. The OTU sequences were assigned taxonomies using SINTAX [29] and 16S RDP training set v19 as a reference [30]. The taxonomic structure of the microbiome was calculated by the ratio of the number of taxon-specific sequence reads to the total number of sequences reads, yielding the relative abundance of each taxa in percentage.

## 3. Results

### 3.1. Clinical Characteristics of Patients

The median age of the IE patients recruited in this study was 37 years; males predominated among the studied patients. In two patients, both the aortic and mitral heart valves were removed during heart valve replacement surgeries. Aortic and mitral heart valves accounted for 90% of the total number of valves removed due to IE, and only one heart valve was removed from the tricuspid position. Preoperative antibacterial therapy was administered to all patients recruited in the study. As a result of routine blood culture, the IE etiology was established in only 25% of cases, and Gram-positive *Streptococcus gordonii* and *Enterococcus faecalis* were identified. The full characteristics of the studied patients are presented in Table 1.

### 3.2. Histopathological Characteristics

**Figure 1:**
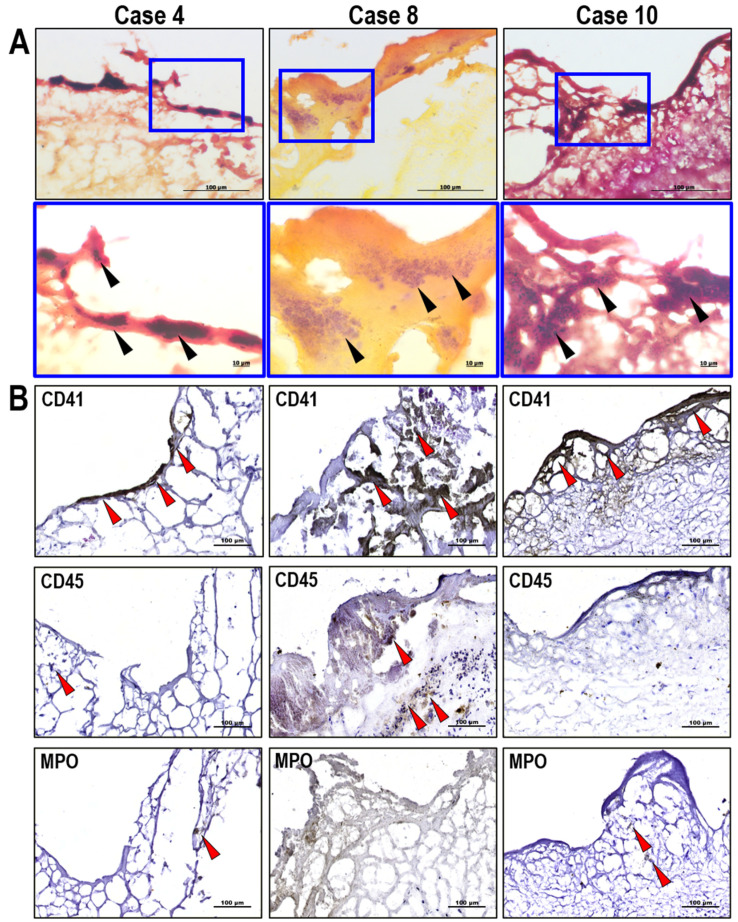
Results of the histopathological analysis of the infective endocarditis-affected native heart valves. (**A**) Gram staining visualization of bacteria in the leaflets of the affected heart valves; the arrows indicate Gram-positive (blue staining) and Gram-negative (red staining) bacterial colonies localized within fibrin deposits (red/orange staining). (**B**) Immunotyping of cells in the microthrombi localized on the leaflet surface in the areas with bacterial invasion; the arrows indicate positively stained cells.

Gram staining of histological sections revealed bacterial cells in only three of the studied native heart valves—cases 4, 8, and 10 (Figure 1A). Analysis of bacterial morphology and staining patterns showed that Gram-positive cocci were present in cases 4, 8, and 10, and Gram-negative bacilli were detected in only cases 4 and 10. It should be noted that cocci and bacilli formed mixed colonies in the IE-affected native heart valves. The detected bacteria were localized on the atrial surface at the leaflet’s free edge (cases 4 and 10) or on the ventricular surface at the leaflet’s base (case 8) within fibrin deposits. CD41 antibody staining showed that bacterial colonies were co-localized with microthrombi containing single leucocytes (CD45^+^ cells). Analysis of the cell nucleus morphology and MPO antibody staining confirmed that the detected leucocytes were represented by segmented neutrophils (Figure 1B).

### 3.3. Taxonomic Structure of Microbiome

**Figure 2:**
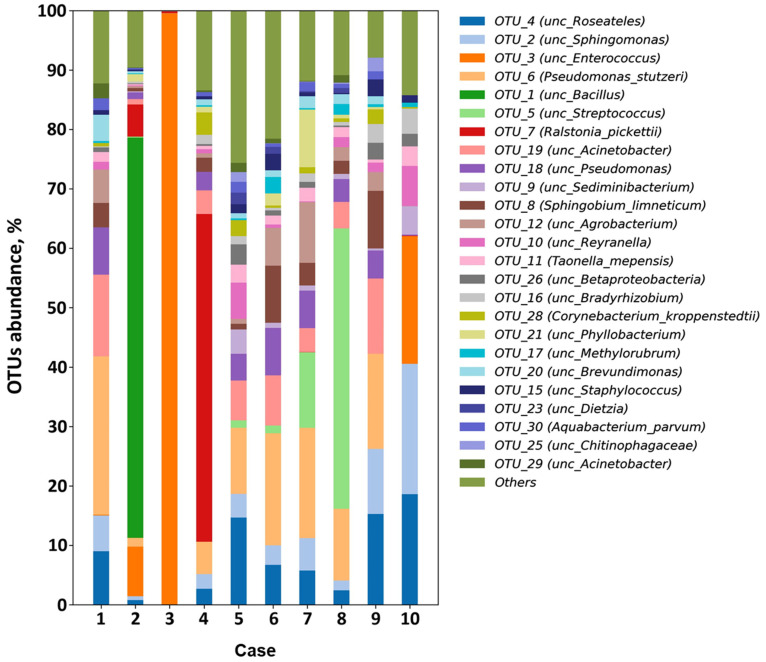
The infective endocarditis-affected native heart valve microbiome (OTUs, operational taxonomic units).

As a result of 16S rRNA metabarcoding, the bacterial agents were identified in all of the studied native heart valves obtained from IE patients (Figure 2).

After applying the cut-off criterion (OTU abundance > 5%), the major pathogens were selected. It was shown that the most frequent bacterial genera were *Pseudomonas* (70% of the studied heart valves) and *Roseateles* (60%). *Acinetobacter* (40%), *Sphingomonas* (40%), *Enterococcus* (30%), *Reyranella* (20%), *Sphingobium* (20%), *Streptococcus* (20%), *Agrobacterium* (20%), *Ralstonia* (10%), and *Bacillus* (10%) were detected in less than half of the studied samples. Polymicrobial contamination was detected in 90% of cases; in one sample (case 3), only *Enterococcus* spp. was identified. Analysis of the IE-affected native heart valves removed from different positions of the same patient (cases 2, 3, 7, and 8) showed that the aortic valves (cases 2 and 7) were characterized by a higher taxonomic diversity of bacteria compared to the mitral valves (cases 3 and 8). It should be noted that the blood culture results were confirmed by 16S rRNA metabarcoding of the heart valves in 100% of cases (Table 2). The full list of identified pathogens is presented in Supplementary Table S1.

## 4. Discussion

Recent epidemiological studies have demonstrated increasing IE incidence and mortality due to its complications, so improving IE prevention, diagnosis, and treatment strategies are extremely important tasks for modern medicine [31]. Correct pathogen identification is critical for the selection of appropriate antibiotic therapy, which in turn reduces the risk of prosthetic heart valve bacteremia [18]. However, the effective identification of IE pathogens using routine blood culture is challenging due to the high percentage of false negative results [13]. The application of modern methods, such as histopathological analysis of operatively excised heart valves affected by IE and 16S rRNA metabarcoding, allows not only to more accurate identify pathogens, even in the cases of blood culture-negative endocarditis, but also to perform in-depth studies of IE pathogenesis, etiology, and epidemiology.

In the present research, histopathological analysis of the native heart valves obtained from IE patients revealed bacteria in 30% of the affected valves. Microorganisms were detected in microthrombi localized in areas characterized by an absent or fragmented endothelial monolayer. These formations were small and could be only observed microscopically; no bacterial vegetations were detected. We also discovered only minor infiltration of microthrombi by single neutrophils. The observed histopathological picture is consistent with the modern concepts of IE pathogenesis [32]. Microorganisms circulating in the blood can attach to the endothelial monolayer of heart valves directly or using activated platelets and enclose themselves in a protective extracellular matrix containing various cellular and molecular components (platelets, fibrin and other blood proteins, bacterial polysaccharides, and extracellular DNA) [6,33]. Subsequent leukocyte infiltration fails to prevent IE progression: activated immune and endothelial cells express tissue coagulation factor to counteract the infectious process, thereby provoking additional fibrin deposition and thrombus growth. In this way, the coagulation system inadvertently creates a “sanctuary” for bacteria, protecting them from recognition and destruction by the immune system instead of their elimination. Previous histological studies showed that IE lesions predominantly consist of fibrin-enmeshed bacterial colonies, often completely devoid of leukocytes [34,35], which is entirely consistent with our results.

It is known that bacteria from the genera *Staphylococcus*, *Streptococcus*, and *Enterococcus* and HACEC organisms are the most common causative agents of IE [13,36]. Moreover, various intracellular bacteria and opportunistic pathogens can initiate an inflammatory process leading to blood culture-negative endocarditis [18,37]. It should be noted that its identification by routine methods is difficult or even impossible. Nowadays, 16S rRNA metabarcoding is the most effective method for identification of the causative agents of IE, especially in patients with blood culture-negative endocarditis [38].

As a result of 16S rRNA metabarcoding, we found no *Staphylococcus* spp. in the analyzed heart valves, which is contrary to the published data [18,24,39]. The well-known IE-related pathogens (streptococci and enterococci) were identified in only 20% and 30% of the analyzed native heart valves, respectively. This is probably due to effective antibiotic therapy against the well-known IE-associated pathogens prior to valve replacement surgery in the studied IE patients. The genus *Enterococcus* is represented by Gram-positive bacteria that occupy a leading position among IE-related pathogens. Enterococci, mainly *E*. *faecalis*, cause up to 20% of all IE cases [40] and are associated with some difficulties in treatment, both in native and prosthetic IE, due to the formation of a biofilm that maintains the bacterial colony [41,42]. It should be noted that *E*. *faecalis*-caused IE is characterized by relapses after heart valve replacement that can occur even after discontinuation of antibiotic therapy in IE patients [43,44].

In our research, *Pseudomonas* spp. was identified in most of the analyzed IE-affected native heart valves. *Pseudomonas* spp. is a widely distributed Gram-negative bacterium that was identified as an opportunistic pathogen in clinical conditions [45]. Only a few clinical reports described *P*. *stutzeri*, *P*. *aeruginosa*, and *P*. *luteola* as pathogens that can cause IE, characterized by a high mortality rate mainly in intravenous drug addicts and immunocompromised patients [40,46,47,48,49,50]. The rarity of *Pseudomonas* spp.-caused IE can be explained by the low infectivity and pathogenicity of this bacteria, as well as by ineffective routine diagnostic methods for its identification [48].

*Acinetobacter* spp., identified in 40% of the analyzed heart valves, are Gram-negative bacteria that rarely cause IE. According to the clinical studies, *A*. *baumannii* and *A*. *calcoaceticus* are the main IE causative agents [51]. *Acinetobacter* spp.-caused IE is characterized by a resistance to antimicrobial drugs [52] and a high mortality rate [53].

*Sphyngomonas paucimobilis* is an opportunist Gram-negative bacillus characterized by multidrug resistance and is implicated in a wide variety of both community- and hospital-acquired infections [54]. Some clinical reports demonstrated that *S*. *paucimobilis* is associated with both prosthetic and native valve IE [55,56,57,58]. Infections caused by *S*. *paucimobilis* are rarely fatal due to a low virulence of this bacteria [59].

In addition, we discovered single cases of bacterial invasion by *Ralstonia* spp., *Bacillus* spp., and *Agrobacterium* spp. in the studied heart valves. *Ralstonia pickettii*, an aerobic, Gram-negative, oxidase-positive bacillus, has been recently described as an opportunistic pathogen in patients with acquired (HIV) or pharmaceutical immunosuppression [60]. *R*. *pickettii* can survive in a wide temperature range (15–42 °C) and form a biofilm that is resistant to the host immune response and a number of antibiotics [61,62]. Nowadays, there is no standardized treatment protocol for *R*. *pickettii*-caused infection due to its differential susceptibility to antibiotics. In the presented study, *Ralstonia* spp. was identified in the heart valve obtained from an IE patient concomitated by HIV and hepatitis C, which is consistent with the literature data.

*Bacillus* spp. are Gram-positive facultative aerobes that can be found in the environment, food, and the intestinal microflora [63]. It was also shown that some species from the genus *Bacillus* mainly affect mitral valves and are associated with high mortality in patients with prosthetic valve IE [64]. It is known that *Bacillus* spp. causes IE in patients with intravenous drug use, venous catheters, prosthetic heart valves, malignant neoplasms, and immunosuppression [64,65].

*Agrobacterium radiobacter* (*Rhizobium radiobacter*) has been described as a Gram-negative opportunistic pathogen [66] that can cause both prosthetic and native heart valve endocarditis in patients with intravascular catheters [67,68,69,70,71]. It was shown that patients with malignancy or HIV characterized by neutropenia, leukopenia, and low CD4^+^ lymphocyte count had a higher risk of *A*. *radiobacter*-caused IE.

Interestingly, that aortic heart valves were characterized by a higher taxonomic diversity of bacteria compared to the valves removed from the mitral position of the same patient. This can be explained by the fact that aortic valves are constantly subject to high pressure, while mitral valves are never exposed to high pressure [72]. The constant action of high pressure on aortic valves likely results in damage to the endothelial monolayer lining the valve surface, which increases the risk of bacterial adhesion and its penetration into the valve tissue.

Here, we identified *Roseateles* spp., *Reyranella* spp., and *Sphingobium* spp. in the native heart valves obtained from IE patients for the first time. There are currently no clinical case reports describing the association of these bacteria with IE, so their role in the development of this pathology is questionable and requires further study.

It should be noted that the present study is limited by its small sample size and the preoperative antibiotic therapy of patients recruited to the study.

## 5. Conclusions

According to clinical studies using standard microbiological approaches, infections by single bacterial species are described in 99% of all IE cases. The identified bacteria are usually considered to be the causative agents of IE, and appropriate antibiotic therapy aimed primarily at *Staphylococcus* spp., *Streptococcus* spp., *Enterococcus* spp., and HACEC organisms is selected. At the same time, a number of opportunistic microorganisms that cannot be detected by routine laboratory tests and are not eliminated during antibiotic therapy, as well as some pathogenic microorganisms, which are probably represented by antibiotic-resistant strains, have been identified in IE-affected heart valves. Our findings demonstrated that native heart valves removed from patients with blood culture-negative endocarditis were characterized by a large number of opportunistic pathogens that could not be detected by routine non-invasive diagnostic methods. This indicates that the residual bacteremia in patients administered preoperative antibiotic therapy directed mainly against well-known IE pathogens can lead to the development of prosthetic IE in the postoperative period, followed by reoperation, deterioration of the patient’s quality of life, and increased risk of mortality. The obtained results show the importance of 16S rRNA metabarcoding of heart valves removed due to IE in clinical practice and the need for an in-depth study of the taxonomic diversity of IE-affected heart valves using high-throughput methods.

## Figures and Tables

**Table 1:** Clinical characteristics of the studied infective endocarditis patients.

Indicator		Value
Clicinal-Demografic Indicators		
Gender	Males, *n* (%)	5 (62.5%)
	Females, *n* (%)	3 (37.5%)
Mean age, Median (Q1; Q3)		37 (33; 57)
Left ventricular ejection fraction, Mean ± SD		61.88 ± 11.74
Preoperative antibiotic therapy, *n* (%)		8 (100%)
Intravenous drug use, *n* (%)		1 (12.5%)
Affected heart valve (*n* = 10)	Aortic, *n* (%)	4 (40%)
	Mitral, *n* (%)	5 (50%)
	Tricuspid, *n* (%)	1 (10%)
**Laboratory Indicators**		
Leucocites (10^9^/L), Mean ± SD		8.46 ± 3.16
Neutophils (10^9^/L), Mean ± SD		5.57 ± 2.61
C-reactive protein (mg/L), Mean ± SD		9.58 ± 12.51
Erythrocyte sedimentation rate (mm/h), Mean ± SD		23.89 ± 22.09
**Results of Blood Culture for Pathogen Identification**		
Positive, *n* (%)		2 (25%)
Negative, *n* (%)		6 (75%)

**Table 2:** Taxonomic diversity of bacteria identified in patients with infective endocarditis.

Patient	Case	Affected Heart Valve	Identified Bacteria		
			Blood Culture	Gram Stain of Heart Valves	16S rRNA Metabarcoding *
1	Case 1	Aortic	Sterile	Negative	*Pseudomonas stutzeri* (27%) unc_*Acinetobacter* (14%) unc_*Roseateles* (9%) unc_*Pseudomonas* (8%) unc_*Sphingomonas* (6%)
2	Case 2	Aortic	*Enterococcus* *faecalis*	Negative	unc_*Bacillus* (67%) unc_*Enterococcus* (8.3%)
	Case 3	Mitral	*Enterococcus* *faecalis*	Negative	unc_*Enterococcus* (100%)
3	Case 4	Aortic	Sterile	Bacilli, cocci	*Ralstonia pickettii* (55%) *Pseudomonas stutzeri* (5.5%)
4	Case 5	Mitral	Sterile	Negative	unc_*Roseateles* (15%) *Pseudomonas stutzeri* (11%) unc_*Acinetobacter* (6.7%) unc_*Reyranella* (6.1%)
5	Case 6	Mitral	Sterile	Negative	*Pseudomonas stutzeri* (19%) *Sphingobium limneticum* (9.6%) unc_*Acinetobacter* (8.4%) unc_*Pseudomonas* (8.0%) unc_*Roseateles* (6.7%) unc_*Agrobacterium* (6.4%)
6	Case 7	Aortic	*Streptococcus* *gordonii*	Negative	*Pseudomonas stutzeri* (19%) unc_*Streptococcus* (13%) unc_*Agrobacterium* (10%) unc_*Pseudomonas* (6.3%) unc_*Roseateles* (5.8%) unc_*Sphingomonas* (5.5%)
	Case 8	Mitral	*Streptococcus* *gordonii*	Cocci	unc_*Streptococcus* (47%) *Pseudomonas stutzeri* (12%)
7	Case 9	Tricuspid	Sterile	Negative	*Pseudomonas stutzeri* (16%) unc_*Roseateles* (15%) unc_*Sphingomonas* (11%) unc_*Acinetobacter* (13%) *Sphingobium limneticum* (9.7%)
8	Case 10	Mitral	Sterile	Bacilli, cocci	unc_*Enterococcus* (22%) unc_*Sphingomonas* (22%) unc_*Roseateles* (19%) unc_*Reyranella* (6.8%)

* The major pathogens with OTU abundance > 5% (OTUs, operational taxonomic units).

## Data Availability

The read data were submitted to the Sequence Read Archive (SRA) under study accession number PRJNA1194321 (https://www.ncbi.nlm.nih.gov/bioproject/PRJNA1194321) (accessed on 14 January 2025).

## Supplementary Materials

The following supporting information can be downloaded at: https://www.mdpi.com/article/10.3390/biomedicines13010245/s1, Table S1: The full list of identified using 16S rRNA metabarcoding pathogens.
