# Supplementary material for: Bacterial Diversity in Native Heart Valves in Infective Endocarditis
Source: Biomedicines. 2025 Jan 20;13(1):245. doi: 10.3390/biomedicines13010245 (PMC11762347; doi:10.3390/biomedicines13010245)
Supplement: Supplementary file 1 [file biomedicines-13-00245-s001.zip › biomedicines-3397741-supplementary.pdf]

Supplementary Table S1. The full list of identified using 16S rRNA metabarcoding pathogens

**OTUs Abundance, %**	**Case 1**	**Case 2**	**Case 3**	**Case 4**	**Case 5**	**Case 6**	**Case 7**	**Case 8**	**Case 9**	**Case 10**
**OTU_4 (unc_Roseateles)**	**9.0**	0.8	0	2.7	**15**	**6.7**	**5.8**	2.4	**15**	19
**OTU_2 (unc_Sphingomonas)**	**6.0**	0.6	0	2.5	4.0	3.3	**5.5**	1.7	**11**	22
**OTU_3 (unc_Enterococcus)**	0.1	**8.3**	**100**	0	0	0	0	1.6E-03	0	22
**OTU_6 (Pseudomonas_stutzeri)**	**27**	1.5	1.0E-02	**5.5**	**11**	**19**	**19**	**12**	**16**	2.5E-03
**OTU_1 (unc_Bacillus)**	0	**67**	0	0	0	0	0	0	0	0
**OTU_5 (unc_Streptococcus)**	0	0.2	0	0	1.3	1.3	**13**	**47**	0	0
**OTU_7 (Ralstonia_pickettii)**	0	**5.4**	0.3	**55**	0	1.7E-03	0.1	0	0	0.1
**OTU_19 (unc_Acinetobacter)**	**14**	0.9	4.8E-03	4.0	**6.7**	**8.4**	4.0	4.5	**13**	5.0E-03
**OTU_18 (unc_Pseudomonas)**	**8.0**	1.1	9.5E-03	3.1	4.5	**8.0**	**6.3**	3.8	4.7	0.2
**OTU_9 (unc_Sediminibacterium)**	0	0.2	4.8E-03	0	4.1	0.8	0.9	0.8	0.4	4.8
**OTU_8 (Sphingobium_limneticum)**	4.1	0.5	0	2.4	1.0	**9.6**	3.8	2.2	**9.7**	0
**OTU_12 (unc_Agrobacterium)**	5.7	0.3	9.5E-03	0.7	0.8	**6.4**	**10**	2.3	3.2	5.0E-03
**OTU_10 (unc_Reyranella)**	1.3	0.1	0	0.7	**6.1**	0.6	0.1	1.7	1.6	6.8
**OTU_11 (Taonella_mepensis)**	1.7	0.2	4.8E-03	0.6	3.0	1.5	2.3	1.7	0.5	3.3
**OTU_26 (unc_Betaproteobacteria)**	0.8	0.1	0	0.3	3.4	0.9	1.0	0.3	2.8	2.1
**OTU_16 (unc_Bradyrhizobium)**	0.2	0.3	2.0E-02	1.5	1.4	0.4	1.4	0.6	3.2	4.2
**OTU_28 (Corynebacterium_kroppenstedtii)**	0.6	2.0E-02	4.8E-03	3.8	2.7	0.4	1.1	0.6	2.5	0.3
**OTU_21 (unc_Phyllobacterium)**	0	1.3	0.1	1.0	0	2.0	9.7	0.6	0.4	1.0E-02
**OTU_17 (unc_Methylorubrum)**	0.3	0.1	0	0.3	0.3	2.8	0.3	1.8	0.5	0.7
**OTU_20 (unc_Brevundimonas)**	4.4	0.3	0	1.0	0.9	1.1	2.0	1.7	1.4	0
**OTU_15 (unc_Staphylococcus)**	0.8	0.3	0	0.5	1.5	2.8	0.7	0.2	2.8	1.3
**OTU_23 (unc_Dietzia)**	0	0	0	0	1.9	1.2	0.2	0.8	0.1	5.0E-03

**OTU_30 (Aquabacterium_parvum)**	2.0	0.3	0	0.7	1.8	0.6	1.5	0.7	1.3	0
**OTU_25 (unc_Chitinophagaceae)**	0	7.1E-03	0	0	1.7	0.1	0.1	0.2	2.3	0
**OTU_29 (unc_Acinetobacter)**	2.5	0.1	0	0.3	1.6	0.8	0.1	1.3	0	0
**OTU_35 (unc_Micrococcus)**	0.7	0.1	0	0.6	0.6	0	0.8	0.9	0	0
**OTU_36 (unc_Delftia)**	0.6	0.1	0	0.6	1.2	2.1	0.3	0.5	0.3	0
**OTU_27 (unc_Stenotrophobacter)**	0	0	0	0	0	0.1	0	0	0	5.4
**OTU_39 (unc_Streptococcus)**	0.3	0.2	0	0.0	0.1	0.4	0.1	1.0E-02	0.2	0
**OTU_32 (unc_Sphingopyxis)**	0.7	0.2	0	0.8	1.6	0	0.5	0.5	0	0
**OTU_51 (unc_Priestia)**	0	0	0	4.1	2.0E-02	0	0	0	0	0
**OTU_40 (unc_Corynebacterium)**	0	0.2	0	0	0.7	0.5	0.7	0	0	0
**OTU_37 (unc_Paracoccus)**	0.4	5.3E-03	0	0.1	0.3	0.0	0	0	0.3	0
**OTU_31 (unc_Bosea)**	0.1	0.1	0	5.3E-03	0.1	0.1	0	0.1	0	2.9
**OTU_33 (unc_Methylobacterium)**	1.1	0	0	0	0.8	1.8	0	0	0.1	0
**OTU_34 (unc_Pedomicrobium)**	0	0	0	0	0	0	0.1	8.1E-03	0.1	1.3
**OTU_38 (Lawsonella_clevelandensis)**	0	0	0	0.6	0.7	1.7	0	0	0	0
**OTU_71 (unc_Blastomonas)**	1.4	0.2	4.8E-03	0.2	0.5	0.1	0.1	0.4	0	0
**OTU_22 (unc_Enterobacteriaceae)**	0	2.8	0	1.0E-02	0	0	0	0.1	0	0
**OTU_64 (Sphingobacterium_psychroaquaticum)**	0.7	0.1	0	0.3	0	0	1.7	0	0	0
**OTU_61 (unc_Sphingomonas)**	0	0.2	0	0.4	0.3	0.9	0	0	0.2	0
**OTU_42 (unc_Acidibacter)**	0.1	0	0	0	1.1	0	0	0.4	1.0E-02	0.4
**OTU_41 (unc_Acidovorax)**	4.6E-03	0.1	0	0.1	1.1	1.0	0.1	0	0	0
**OTU_44 (unc_Brachybacterium)**	0	0.2	0	0.2	0	1.8	0	0.1	0	0
**OTU_55 (unc_Kocuria)**	0	0.2	0	0	0	0.2	0	0	0	1.7
**OTU_50 (unc_Pseudomonas)**	0	0	0	0	0	1.2	0.2	0.2	0.2	0
**OTU_46 (unc_Corynebacterium)**	0.1	0	0	0	0	0	0.6	1.1	0	0
**OTU_63 (unc_Paraburkholderia)**	0.7	0	0	0.1	0.1	0.2	0.3	0.3	0	0
**OTU_45 (unc_Nevskiaceae)**	0	0	0	0.1	1.2	0	0	1.0E-02	0	0
**OTU_74 (Cutibacterium_acnes)**	4.6E-03	0	0	1.0E-02	0.1	0.0	0	0	0	0
**OTU_60 (unc_Brevibacterium)**	0	0	0	0.1	0	0.7	0	0	0	0

**OTU_68 (Truepera_radiovictrix)**	0	0	0	0	0	0	0	0	1.5	0
**OTU_47 (unc_Phenylobacterium)**	0	0	0	0	0	0	0.0	0.2	0.6	0.6
**OTU_57 (unc_Sphaerobacteraceae)**	0.7	1.8E-03	0	0	0	0.6	0	0	0.2	0
**OTU_79 (unc_Stenotrophomonas)**	0.2	0.3	0	0	0.5	0.2	1.0E-02	0.2	0	0
**OTU_53 (unc_Planococcus)**	0.1	0	0	0	0	1.3	0	0	0	0
**OTU_92 (unc_Brevibacterium)**	0	0	0	0.3	0.2	0	0.5	0	0.1	0.4
**OTU_69 (unc_Melaminivora)**	0	0	0	0	0	0	0.0	0	0.1	0
**OTU_78 (unc_Caballeronia)**	0	0	0	0	0	0	0	0	0	0
**OTU_67 (unc_Neisseriaceae)**	0	0	0	0.4	0.7	0	0	0	0	0.2
**OTU_109 (unc_Streptomyces)**	0	0.0	0	1.2	0	0	0	0	0	0
**OTU_48 (unc_Caulobacter)**	0.1	0	0	0	1.1	0	0	0	0	0
**OTU_227 (unc_Acinetobacter)**	0.4	0	0	0	0	2.0E-02	0.5	0.2	0.1	0
**OTU_75 (unc_Rubrobacter)**	0	3.5E-03	0	0	0.0	0	0.2	0	0	1.0
**OTU_76 (unc_Acinetobacter)**	0	0	0	0	0.7	0	0	0	0.6	0
**OTU_85 (unc_Prevotellaceae)**	0	0	0	0	0.7	0	0	0	0	0
**OTU_108 (unc_Streptophyta)**	0	3.5E-03	0	0	0.1	0	0.9	0	0.2	0
**OTU_49 (unc_Brevundimonas)**	0	0.2	0	0.4	0.5	0	0	0	0	2.5E-03
**OTU_105 (unc_Glutamicibacter)**	0.8	0	0	0.3	0	0	0	0	0	0
**OTU_119 (Haemophilus_parainfluenzae)**	0	0	0	0	0.7	0	0	0	0	0
**OTU_43 (unc_Brevundimonas)**	0	0	0	0	0	1.0	0	0	0	0
**OTU_95 (Anaerococcus_octavius)**	0	0	0	2.0E-02	0	0	0	0	0	0
**OTU_94 (unc_Cupriavidus)**	0	0	0	0	0	0.9	0	0	0	0
**OTU_62 (unc_Mammaliicoccus)**	0.1	0	0	0	0.8	0	0	0	0	0
**OTU_59 (unc_Faecalibacterium)**	0	0	0	0	0	0	0	0.8	0.1	0
**OTU_54 (Peptoniphilus_duerdenii)**	0	0	0	0	0.8	0	0	0	0	0
**OTU_99 (unc_Gammaproteobacteria)**	0	0	0	0	0	0.8	0	0	0	0
**OTU_104 (unc_Thiopseudomonas)**	0	0	0	0	0	0	0	0	0	0
**OTU_179 (unc_Veillonella)**	0.1	0.1	0	0	0	0	0.6	0	0	0
**OTU_158 (Pedobacter_nutrimenti)**	0	0	0	0	0	0	0.7	0	0	0
**OTU_52 (unc_Hyphomicrobiales)**	0	0	0	0	0	0.7	0	0	0	0

**OTU_96 (Moraxella_osloensis)**	0.1	0.0	0	0.1	0.2	0	0.3	0.1	0	0
**OTU_90 (unc_Proteiniphilum)**	0	0	0	0	0	0	0	0	0	0
**OTU_70 (unc_Janibacter)**	0	0	0	0	0.4	0	0	0.3	0	0
**OTU_81 (unc_Corynebacterium)**	0	0	0	0	0	0	0	0	0	0
**OTU_244 (Haemophilus_haemolyticus)**	0	0	0	0	0.6	0	0	0	0	0
**OTU_84 (Granulicatella_elegans)**	0	0	0	0	0.6	0	0	0	0	0
**OTU_118 (unc_Haemophilus)**	0	0	0	0	0.6	0	0	0	0	0
**OTU_91 (unc_Barrientosiimonas)**	0	0	0	0	0.6	0	0	0	0	0
**OTU_97 (unc_Neisseria)**	0	1.8E-03	0	0	0.6	0	0	0	0	0
**OTU_72 (Anaerococcus_nagyae)**	0	0	0	0	0.6	0	0	0	0	0
**OTU_87 (unc_Neisseriaceae)**	0	0	0	0.1	0	0.3	0	0.2	0	0
**OTU_262 (Abiotrophia_defectiva)**	0.6	0	0	0	0	0	0	0	0	0
**OTU_100 (unc_Anaerobacillus)**	0	3.5E-03	0	0	0	0.5	0.1	0	0	0
**OTU_24 (unc_Escherichia/Shigella)**	0	0.6	0	0	0	0	0	0	0	0
**OTU_206 (Tepidiphilus_margaritifer)**	0	0	0	0	0	0	0.5	0	0	0
**OTU_132 (Cloacibacterium_normanense)**	0	1.8E-03	0	0	0	0	0	0	0.5	0
**OTU_190 (unc_Bacillaceae)**	0.5	3.5E-03	0	0	0	0	0	0	0	0
**OTU_185 (unc_Parazoarcus)**	0	0	0	0	0	0	0	0	0.5	0
**OTU_89 (unc_Brevibacillus)**	0	0	0	0	0	0	0	0.5	0	0
**OTU_98 (unc_Bacillus)**	0	0.1	0	0	0.5	0	0	0	0	0
**OTU_140 (unc_Microbacterium)**	0.1	0	0	0	8.1E-03	0	0	0	2.0E-02	0
**OTU_175 (unc_Pseudoxanthomonas)**	0	0	0	0	0	0	0.4	0	0.2	0
**OTU_82 (unc_Azospirillaceae)**	0	0.1	0	0.3	0	0.2	0	0	0	0
**OTU_123 (unc_Bacteria)**	0.5	0	0	0	0	0	0	0	0	0
**OTU_88 (unc_WPS-2_genera_incertae_sedis)**	0	0	0	0.1	0	0	0	0	0	0
**OTU_130 (unc_Peribacillus)**	0	0.2	0	0.3	0	0	0	0	0	0
**OTU_151 (unc_Rhizobiaceae)**	0	0	0	0	0	0.5	0	0	0	0
**OTU_117 (Comamonas_aquatilis)**	0	0	0	0.4	0.0	0	0	0	0.0	0
**OTU_65 (Corynebacterium_otitidis)**	0	0.5	0	0	0	0	0	0	0	0

**OTU_80 (unc_Gallionellaceae)**	0	0	0	0	0	0	0	0.5	0	0
**OTU_83 (unc_Jeotgalicoccus)**	0	0	0	0	0	0	0	0.5	0	0
**OTU_66 (unc_Anaerococcus)**	0	0	0	0	0	0	0	0.5	0	0
**OTU_13 (Limosilactobacillus_fermentum)**	0	0.4	0	0	0	0	0	0	0	0
**OTU_115 (unc_Chryseobacterium)**	0	0	0	0	0	0	0	0	0	0
**OTU_191 (unc_Acinetobacter)**	0	0	0	0	0	0	0.4	0	0	0
**OTU_93 (Porphyromonas_pasteri)**	0	0	0	0	0.4	0	0	0	0	0
**OTU_73 (Fusobacterium_necrophorum)**	0	0	0	0	0.4	0	0	0	0	0
**OTU_201 (Weizmannia_coagulans)**	0	0	0	0	0	0	0.4	0	0	0
**OTU_106 (unc_Bacillaceae)**	0	0	0	0	0	0	0	0	0	0
**OTU_146 (Bacteroides_uniformis)**	0	0.4	0	0	0	0	0	0	0	0
**OTU_102 (Alloiococcus_otitis)**	0	0.4	0	0	0	0	0	0	0	0
**OTU_164 (Deinococcus_budaensis)**	0	0	0	0	0	0	0	0	0.4	0
**OTU_178 (unc_Comamonadaceae)**	0	0	0	0	4.0E-03	0.0	0	0.0	0.3	0
**OTU_139 (unc_Rubellimicrobium)**	0	0	0	0	0	0	0	0.3	0	0
**OTU_111 (Tissierella_creatinophila)**	0	0	0	0.3	0	0	0	0	0	0
**OTU_116 (Hoylesella_nanceiensis)**	0	0	0	0	0.3	0	0	0	0	0
**OTU_122 (unc_Helcococcus)**	0	0	0	0	0	0.3	0	0	0	0
**OTU_101 (unc_Aliterella)**	0	0	0	0	0	0.3	0	0	0	0
**OTU_112 (unc_Bacillus)**	0	0.1	0	0	0	0	0	0.2	0	0
**OTU_110 (unc_Isoptericola)**	0	2.0E-02	0	0	0	0	0	0.3	0	0
**OTU_184 (Luteimonas_terrae)**	0	0	0	0	0	0.1	0	0	0.2	0
**OTU_202 (Polynucleobacter_difficilis)**	0	0	0	0	0	0	0.3	0	0	0
**OTU_86 (Terriglobus_roseus)**	0	0	0	0	0	0	0	0.3	0	0
**OTU_148 (unc_Coleofasciculales)**	0.3	0	0	0	0	0	0	0	0	0
**OTU_103 (Ezakiella_coagulans)**	0	0	0	0	0.3	0	0	0	0	0
**OTU_113 (Mesorhizobium_terrae)**	0	0	0	0	0	0	0	0	0	0.3
**OTU_168 (unc_Aquabacterium)**	0.2	0	0	0	0	0	0.0	0.0	0	0
**OTU_136 (Chryseobacterium_hominis)**	0	0	0	0.3	0	0	0	0	0	0

**OTU_114 (unc_Curvibacter)**	0.1	1.8E-03	0	2.0E-02	0.0	0	0	0.2	0	0
**OTU_142 (unc_Ornithinimicrobium)**	0	0	0	0	0	0	0.0	0.2	0	0
**OTU_147 (unc_Herbaspirillum)**	0	0	0	0	0	0.3	0	0	0	0
**OTU_125 (Finegoldia_magna)**	0	0.1	0	0	0.2	0	0	0	0	0
**OTU_188 (Flaviflexus_salsibiostraticola)**	0	0	0	0	0	0.0	0	0	0.2	0
**OTU_134 (unc_Mycobacterium)**	0	3.5E-03	0	0	0.0	0.2	0.1	0	0	0
**OTU_127 (unc_Comamonas)**	0	2.0E-02	0	0	0	0	0	0.2	0	0
**OTU_149 (Diaphorobacter_nitroreducens)**	0	0	0	0	0.2	0.1	1.0E-02	0	0	0
**OTU_166 (unc_Pseudonocardia)**	4.6E-03	0	0	0	0	0	0	0	0	0
**OTU_135 (unc_Rubellimicrobium)**	0	0	0	0	0.2	0	0	0	0	0
**OTU_131 (unc_Parvimonas)**	0	0	0	0	0.2	0	0	0	0	0
**OTU_126 (unc_Actinomycetota)**	0	0.2	0	0	0	0	0	0	0	0
**OTU_137 (Cytophaga_aurantiaca)**	0	0	0	0	0.2	0	0	0	0	0
**OTU_195 (unc_Leuconostoc)**	0.2	0	0	0	0	0	0	0	0	0
**OTU_176 (Rothia_dentocariosa)**	0	0	0	0.2	0	0	0	0	0	0
**OTU_120 (Marmoricola_aquaticus)**	0	0	0	0	0	0	0	0.2	0	0
**OTU_177 (unc_Fictibacillus)**	0	0	0	0	0	0.2	0	0	0	0
**OTU_128 (unc_Anaerolineae)**	0	0	0	0	0	0	0	0.2	0	0
**OTU_183 (unc_Phenylobacterium)**	0	0	0	0	0	0	0	0	0.2	0
**OTU_129 (unc_Aureimonas)**	0	0	0	0	0.2	0	0	0	0	0
**OTU_153 (Sandaracinobacteroides_hominis)**	0	0	0	0.1	0	0.0	0	0	0	0
**OTU_160 (unc_Legionella)**	0	0.1	0	0	0	8.3E-03	0.1	1.0E-02	0	0
**OTU_233 (Pseudoneobacillus_rhizosphaerae)**	0	0	0	0	0	0	0	0	0.2	0
**OTU_133 (unc_Granulicella)**	0	0	0	0	0.2	0	0	0	0	0
**OTU_199 (unc_Solirubrobacterales)**	0	0	0	0.2	0	0	0	0	0	0
**OTU_238 (unc_Paraburkholderia)**	0.2	0	0	0	0	0	0	0	0	0
**OTU_172 (unc_Streptococcus)**	0	0	0	0	0.1	0	0	0	0	0
**OTU_269 (Acinetobacter_marinus)**	0	0	0	0	0	0	0.1	0	0	0

**OTU_154 (unc_Gemella)**	0	0	0	0	0.1	0.1	0	0	0	0
**OTU_197 (Rufibacter_ruber)**	0.1	0	0	0	0	0	0	0	0	0
**OTU_138 (unc_Intestinibacter)**	0	0.1	0	0	0	0	0	0	0	0
**OTU_141 (unc_Lachnospiraceae)**	0	0.1	0	0	0	0	0	0	0	0
**OTU_145 (unc_Rubrobacter)**	0	8.9E-03	0	0	0	0.1	0	0	0	0
**OTU_143 (unc_Lactobacillus)**	0	0.1	0	0	0	0	0	0	0	0
**OTU_171 (unc_Deinococcus)**	0	0	0	0.1	0	0	0	0.1	0	0
**OTU_229 (unc_Halomonas)**	0.1	0	0	0	0	0	0	0	0	0
**OTU_162 (unc_Leptolyngbyaceae)**	0	0.1	0	0	0	0	0	0	0	0
**OTU_174 (unc_Alphaproteobacteria)**	0	0	0	0	0.1	0	0	0	0	0
**OTU_152 (Porphyromonas_pasteri)**	0	0	0	0	0.1	0	0	0	0	0
**OTU_159 (Patulibacter_minatonensis)**	0	0.1	0	0	0	0	0	0	0	0
**OTU_180 (unc_Actinomadura)**	0	0	0	0	0.1	0	0	0	0	0
**OTU_157 (unc_Corynebacterium)**	0	0.1	0	0	0	0	0	0	0	0
**OTU_165 (unc_Peptoniphilus)**	0	0	0	0	0.1	0	1.0E-02	0	0	0
**OTU_194 (unc_Bacteria)**	0	0	0	0	0.1	0	0	0	0	0
**OTU_228 (unc_Dietzia)**	0	0	0	0	0	0	0.1	0	0	0
**OTU_212 (Sphingobacterium_daejeonense)**	0	0	0	0	0	0	0	0	0.1	0
**OTU_182 (unc_Bacteria)**	0	0.1	0	0	0	0	0	0	0	0
**OTU_268 (unc_Dyadobacter)**	0	0	0	0	0	0	0.1	0	0	0
**OTU_241 (Cumulibacter_manganitolerans)**	0	0	0	0	0	0	0	0	0	0
**OTU_217 (Nakamurella_aerolata)**	0	0	0	5.3E-03	0	0	0	0	0	0
**OTU_231 (unc_Streptococcus)**	0	0	0	0	0.1	0	0	0	0	0
**OTU_259 (unc_Conexibacter)**	0	0	0	0	0	0	0	0	0.1	0
**OTU_14 (unc_Listeria)**	0	0.1	0	0	0	0	0	0	0	0
**OTU_207 (unc_Acetobacteraceae)**	0	0.1	0	0	0	0	0	0	0	0
**OTU_193 (unc_Spartobacteria_genera_incertae_sedis)**	0	0	0	0	0.1	0	0	0	0	0
**OTU_196 (Prevotella_amnii)**	0	0	0	0	0	0	0	0	0	0.1
**OTU_163 (unc_Aeromonas)**	0	0	0	0	0	0	0.1	0	0	0
**OTU_167 (unc_Bacteria)**	0	0	0	0	0	0	0	0.1	0	0
**OTU_169 (Akkermansia_muciniphila)**	0	0.1	0	0	0	0	0	0	0	0

**OTU_261 (Atopostipes_suicloacalis)**	0	0	0	5.3E-03	0	0	0	0	0.1	0
**OTU_225 (unc_Sphingopyxis)**	0	2.0E-02	0	0	0.0	0	0	0	0	0
**OTU_189 (unc_Blastococcus)**	1.0E-02	0	0	0	0	0	0	0.0	0	0
**OTU_208 (unc_Gemmiger)**	0	0.1	0	0	0	0	0	0	0	0
**OTU_232 (unc_Nocardioides)**	0	0.1	0	0	0	0	0	0	0	0
**OTU_187 (unc_Cytophagales)**	0	0.1	0	0	0	0	0	0	0	0
**OTU_203 (Phocaeicola_plebeius)**	0	0.1	0	0	0	0	0	0	0	0
**OTU_243 (unc_Bacillaceae)**	0	0.1	0	0	0	0	0	0	0	0
**OTU_230 (unc_Mobiluncus)**	0	0	0	0	0.0	0	0	0	0	0
**OTU_219 (unc_Prevotella)**	0	0	0	0	0.0	0	0	0	0	0
**OTU_216 (unc_Collinsella)**	0	0.0	0	0	0	0	0	0	0	0
**OTU_242 (unc_Alkalibacterium)**	0	0	0	0	0	0.0	0	0	0	0
**OTU_223 (unc_Muribaculaceae)**	0	0	0	0	0.0	0	0	0	0	0
**OTU_209 (Asticcacaulis_excentricus)**	0	0	0	0	0.0	0	0	0	0	0
**OTU_186 (unc_Rhodococcus)**	0	0	0	0	0	0	0	0	5.8E-03	0.0
**OTU_220 (unc_Brevibacterium)**	0	0	0	0	0	0	0	0.0	0	0
**OTU_264 (Zea_mays)**	0	0	0	0.0	0	0	0	0	0	0
**OTU_234 (unc_Rheinheimera)**	0	0	0	0.0	0	0	0	0	0	0
**OTU_249 (unc_Gp3)**	0	0	0	0	0	0	0	0	2.0E-02	7.5E-03
**OTU_237 (unc_Acidobacteriaceae)**	0	0	0	0	0.0	0	0	0	0	0
**OTU_248 (Sutterella_massiliensis)**	0	2.0E-02	0	0	0	0	0	0	0	0
**OTU_258 (unc_Actinobacteria)**	0	0	0	0	0	0	2.0E-02	0	0	0
**OTU_252 (unc_Ruminococcus)**	0	2.0E-02	0	0	0	0	0	0	0	0
**OTU_257 (Lachnospira_eligens)**	0	2.0E-02	0	0	0	0	0	0	0	0
**OTU_255 (unc_Bacteria)**	0	0	0	0	0	0	0	0	2.0E-02	0
**OTU_222 (unc_Porphyromonas)**	0	0	0	0	1.0E-02	0	0	0	0	0
**OTU_254 (unc_Paenisporosarcina)**	0	7.1E-03	0	0	0	0	0	0	0	0
